# Papillary Fibroelastoma Originating From the Coumadin Ridge: An Unusual Location of a Primary Cardiac Tumor

**DOI:** 10.1016/j.jaccas.2026.107723

**Published:** 2026-04-01

**Authors:** Joud Fahed, Wongelawit Zerihun, Kaushal Sigdel, Matthew Voss, Jesse A. Doran

**Affiliations:** a Department of Internal Medicine, Ascension Saint Agnes Hospital, Baltimore, Maryland, USA; b Department of Cardiology, Ascension Saint Agnes Hospital, Baltimore, Maryland, USA

**Keywords:** congenital heart defect, echocardiography, imaging

## Abstract

**Background:**

Papillary fibroelastomas are rare, benign cardiac tumors with potential for systemic embolization. Although typically valvular, they may arise in unusual locations and are often detected incidentally during cardiac imaging.

**Case Summary:**

A 76-year-old woman with chronic kidney disease and severe aortic stenosis presented with progressive fatigue and dyspnea. Preoperative imaging for aortic valve replacement revealed a left atrial mass. Transesophageal echocardiography demonstrated a mobile, gelatinous lesion arising from the Coumadin ridge. The mass was surgically excised concurrently with bioprosthetic aortic valve replacement. Histopathology confirmed papillary fibroelastoma.

**Discussion:**

Nonvalvular papillary fibroelastomas are uncommon and may be overlooked. Their mobility confers significant embolic risk, supporting surgical removal, particularly when cardiac surgery is already planned.

**Take-Home Messages:**

Papillary fibroelastomas can occur in rare, nonvalvular locations. Careful imaging is essential for diagnosis and risk assessment. Surgical excision is effective and should be considered to prevent embolic complications.

## Visual Summary

**Figure:**
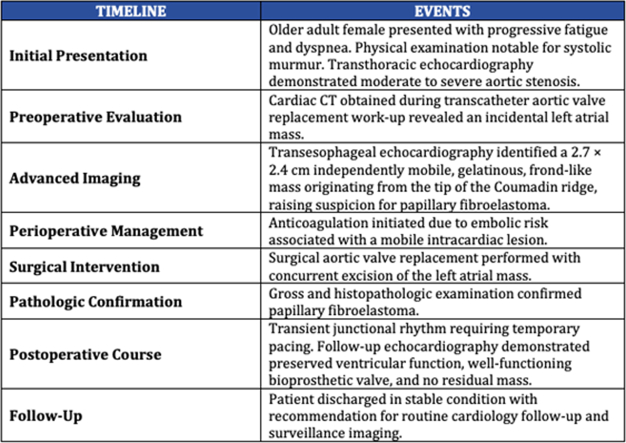

Primary heart tumors are very rare, occurring in about 0.02% of cases, while tumors that spread to the heart from other areas are more common.^1^ Nearly half of all benign heart tumors are myxomas, usually arising from the interatrial septum into the left atrium, while lipomas, papillary fibroelastomas (PFEs), and rhabdomyomas each make up about 10%; other types like fibromas, hemangiomas, teratomas, mesotheliomas, and very rare tumors such as granular cell tumors, neurofibromas, and lymphangiomas are less common.^2^

**Take-Home Messages**

• Cardiac tumors including fibroelastoma can occur in rare, atypical locations including Coumadin ridge.
• Our case highlights the necessity of a multimodal comprehensive cardiac imaging approach, and awareness of atypical locations for surgical resection provides both diagnostic and therapeutic solutions.

Most of the cardiac tumors are found incidentally during evaluation for other conditions.^3^ The tumor's location determines the clinical presentation rather than its histopathology, leading to a wide range of symptoms such as circulatory obstruction, arrhythmias, and embolic phenomena.^3^^,^^4^ Fibroelastomas most commonly originate on the mitral or aortic valves, but less often, they can also be found on the tricuspid valve, ventricular septum, or atrial septum.^2^^,^^5^

This article presents a unique case of a left atrial mass originating from the coumadin ridge identified during preoperative assessment for cardiac surgery, highlighting the diagnostic and management challenges associated with cardiac tumors.

**Visual Summary:**
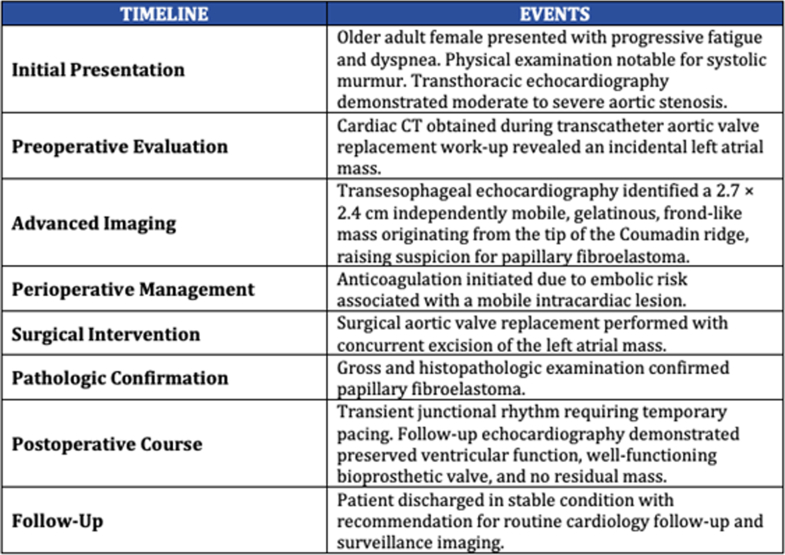
Timeline of the Case

## Case Presentation

A 70-year-old woman with a pertinent past medical history of chronic kidney disease stage II, prior cerebrovascular accident, and severe aortic stenosis presented with progressively worsening fatigue and dyspnea over several months. She had been gradually experiencing worsening shortness of breath, with significant limitations in daily living. Physical examination was notable for a grade 3 ejection systolic murmur with diminished S2. Electrocardiogram showed sinus bradycardia with a heart rate of 54, with no ST/T wave abnormalities. Transthoracic echocardiography was done, which showed normal left ventricular size and function with an estimated ejection fraction of 60% to 65%, moderate to severe aortic stenosis mean pressure gradient across the valve 33 mm Hg, peak aortic valve velocity 54 m/s, and aortic valve area of 1.0 cm^2^.

**Figure 1:**
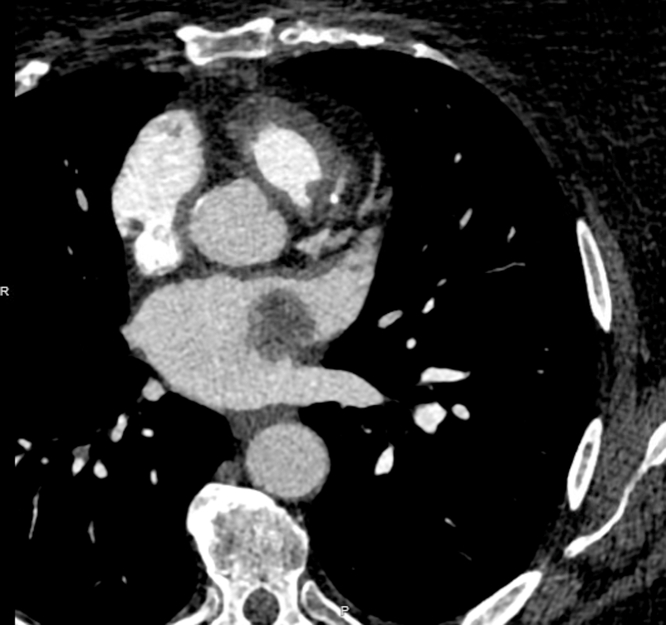
Chest Computed Tomography Showing Filling Defect Measuring Up to 2.2 × 2.1 cm in the Right Atrium Which Appears Pedunculated From the Lateral Atrial Wall

Chest computed tomography (Figure 1) showed an atrial mass. Coronary angiogram showed severe aortic stenosis (43 mm Hg, p 45 m/s) and no obstructive coronary artery disease.

**Figure 2:**
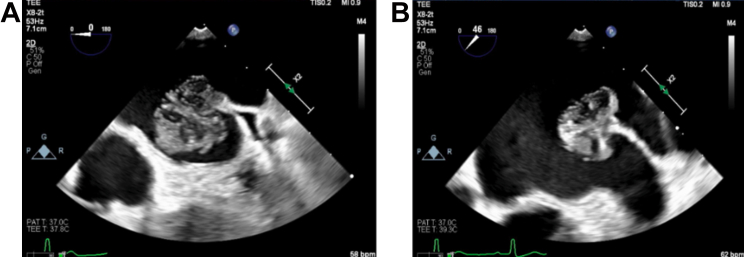
Trans-esophageal Echocardiogram Showing a Well-Circumscribed, Pedunculated Highly Mobile Mass With Irregular Frond Like Texture at the Coumadin Ridge Consistent With a Fibroelastoma (A and B) Transesophageal echocardiogram demonstrating the fibroelastoma on the Coumadin ridge.

**Figure 3:**
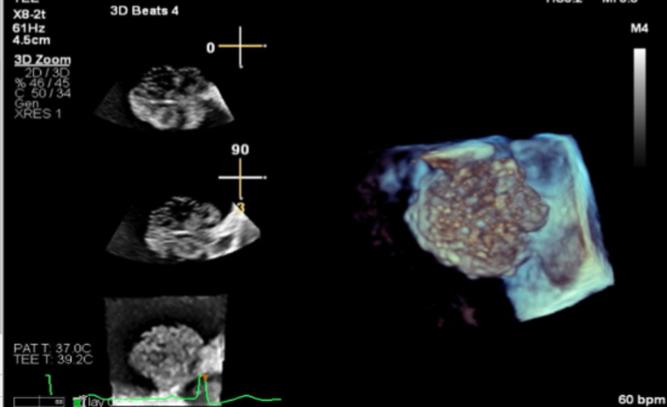
3-Dimensional Image of the Fibroelastoma

She then underwent transesophageal echocardiography for further evaluation, which showed a heterogenous left atrial mass measuring 2.7 × 2.4 cm that originates from the tip of the Coumadin ridge. The mass appeared independently mobile with a “dandelion-like” and gelatinous appearance, and it did not take up diagnostic ultrasound enhancing agent (Figures 2A and 2B, Videos 1A and 1B). Transesophageal echo 3D imaging further characterized the mass to be a stalked mass as opposed to a thrombus or vegetation (Figure 3).

## Management and Outcome

The patient was prophylactically started on anticoagulation with apixaban during the course of preoperative evaluation. She then subsequently underwent surgical aortic valve replacement with a 23-mm Inspiris bioprosthesis. In addition, the left atrial mass was excised through the interatrial septum. The remaining endocardial defect was small and closed primarily using a running 5-0 Prolene suture.

Findings on gross pathologic examination showed a 2.5 × 2.2 × 1.5-cm tan-white, villous, gelatinous soft tissue. The specimen was serially sectioned to reveal a tan-white, firm to gelatinous cut surface, and histological examination was consistent with fibroelastoma.

The postoperative course was initially complicated with junctional rhythm, with 100% pacing requirement. However, around the sixth postoperative day, temporary epicardial wires were able to be removed. Follow-up echocardiogram showed left ventricular ejection fraction in the range of 60% to 65%; normal right and left atrial size and volume with no remnant mass; bioprosthetic aortic valve; peak aortic valve velocity 2 m/s; atrioventricular mean gradient 7 mmHg; Dimensionless Index 0.62; and no aortic regurgitation.

The patient was discharged home in improved condition with discontinuation of anticoagulation and strict recommendation to adhere to ongoing cardiology follow-up and routine health maintenance.

## Discussion

PFEs have recently surpassed cardiac myxomas as the most common benign cardiac tumors. However, they are rarely observed in extravalvular structures of the heart. Fibroblastoma varies 2 to 70 mm in size, with the most common location, over 80%, being on the heart valves, especially left-sided, 36% aortic and 29% mitral, and 9% of cases present with multiple tumors.^6^ These tumors may be symptomatic or discovered incidentally during evaluations for other conditions, as was the case with our patient, where it was identified during routine preoperative assessment for cardiac surgery.

In our patient, the mass was initially found on the Coumadin ridge—an anatomical structure in the left atrium characterized by a prominent ridge of tissue between the left atrial appendage and the left superior pulmonary vein, also known as the left atrial ridge or fold. This location could have easily led to a misdiagnosis as an atrial myxoma. The prevalence of PFEs on the Coumadin ridge is not explicitly documented in the medical literature, with only a few rare case reports describing such occurrences.^7^^,^^8^

The clinical presentation of cardiac tumors is primarily determined by their location rather than their histopathology.^3^ Symptoms can include systemic or pulmonary embolization, circulatory obstruction, heart valve dysfunction, arrhythmias (including heart block), and pericardial effusion with or without tamponade. In some cases, tumors may invade adjacent lung tissue, leading to pulmonary symptoms.

Cardiac tumors may also present with serious complications such as stroke due to embolization, angina, myocardial infarction, sudden death, syncope, or presyncope. Research suggests that the shape and mobility of PFEs may be associated with an increased risk of embolism. In select cases, a robotic surgical approach is feasible, particularly for PFEs located on the mitral valve.^9^

Fibroelastomas are typically pedunculated, mobile, and often flutter or prolapse with cardiac motion. Transesophageal echocardiography is generally more sensitive than transthoracic echocardiography for detecting fibroelastomas,^10^ as it reveals their characteristic frond-like projections extending from a stalked central core.

The type of cardiac tumor could be suggested by its presentation on imaging including echocardiography, cardiac magnetic resonance imaging, or cardiac computed tomography scan by taking into consideration the location, morphology, and functional imaging characteristics and other clinical features including the patient's age.^4^ Information obtained through noninvasive imaging is usually sufficient to determine the need for surgery with the limited data and uncertain benefits of transvenous or transarterial cardiac biopsy for tumor diagnosis.

For resectable-appearing cardiac tumors on imaging, open-technique complete excision with incisional biopsy is usually done because of the risk of embolization or other complications from percutaneous biopsy, whereas for minimal, diffuse, or unresectable tumors, percutaneous biopsy may give valuable information in guiding further management.

The management of PFE depends on the presentation and characteristic of the tumor. Resection of PFEs can be performed safely, with preservation of the native valve and low rates of neurologic events. Operative and long-term outcomes after fibroelastoma resection are excellent.

Surgical intervention is recommended as per American Heart Association and American stroke Association guidelines for symptomatic tumors measuring >1 cm or mobile even though asymptomatic depending on the high risk of embolic events associated with these tumors.^11^ Meanwhile a conservative approach with regular follow-up and echocardiographic monitoring can be considered in small nonmobile tumors and for high-risk surgical candidates with long-term anticoagulation initiation; in some cases, surgical intervention was not pursued.^12^^,^^13^

Following tumor resection, histopathologic examination is essential to confirm the diagnosis, as it helps determine the need for close postoperative monitoring for recurrence or complications. Recurrence rates for PFEs have been reported to be as high as 16%.^9^

PFEs are thought to be neoplastic, hamartomatous, or reactive lesions. New PFEs may develop at or near the site of previous resection due to endocardial injury. In addition, recurrence may result from incomplete tumor removal, the presence of undetected PFEs during surgery, or the formation of a new lesion in a different location.

## Conclusions

This case emphasizes the need to consider uncommon tumor locations, such as the Coumadin ridge, in the evaluation of intracardiac masses. While PFEs are histologically benign, their size and mobility—particularly when exceeding 1 cm—pose a significant risk of embolic complications. The incidental identification of a mobile, gelatinous mass on the Coumadin ridge during aortic valve replacement workup highlights the critical role of comprehensive cardiac imaging and clinical awareness of atypical tumor presentations. Surgical resection served both diagnostic and therapeutic purposes, with histopathological analysis confirming the diagnosis of PFE. This reinforces the necessity of a surgical intervention for high-risk lesions, even in asymptomatic cases. Given the potential for recurrence, ongoing surveillance with serial imaging is essential. This case adds to the growing body of literature emphasizing vigilance in the detection and management of cardiac tumors in uncommon locations.

## Funding Support and Author Disclosures

The authors have reported that they have no relationships relevant to the contents of this paper to disclose.

## Appendix

Video 1 2D Echocardiography With Contrast Enhancement Showing a Mobile Pedunculated Mass Arising Below the Left Atrial Appendage Around the Coumadin Ridge With Frond-Friable Like Overlying Texture (A and B) Transesophageal echocardiographywith contrast-enhanced images showing heterogenous left atrial mass measuring 2.7 × 2.4 cm that originates from the tip of the Coumadin ridge.
